# Seroprevalence of rift valley fever virus and associated risk factors in small ruminants at human-livestock-wildlife interface within Uganda’s conservation areas

**DOI:** 10.1186/s12917-026-05332-y

**Published:** 2026-02-07

**Authors:** Phiona Katushabe, Dennison Kizito, Charity Angella Nassuna, Joseph Kikwabanga Mutyaba, Swaib A. Lule, Gladys Kiggundu Nakanjako, Nackson Babi, Wilber Ssembajjwe, Tonny Kayizi, Milton Bahati, Martin Esau, Stella Atim, Brian Kivumbi, Teddy Nakayiki Muwawu, Deogratius Nteza, Eric Morris Enyel, Alice Namatovu, Stephen Balinandi, Musa Sekamatte, Gladys Kalema-Zikusoka, Patrick Atimnedi, Julius J. Lutwama, Luke Nyakarahuka, Deo B. Ndumu, COHRIE-Uganda team, Erinah Wanyana, Teddy Namagga, David Odongo, Raymond Nviiri, Justine Nassolo, Mercy Haumba, Collins Agaba, Ruth Stephans Nalukwago, Gorreti Mughebwa, Daphine Joy Khayiyi, Caroline Nadiru, Mugga Julius, Senfuka Fred, George Ongodia, Lwanga John, Simon Wekaalo, Ssali Ogwal, Ebenezer Paul, Charity Mutesi, Amina Monero, Caroline Nyamor, Abdulzak Sekamatte, Sam Okware, Musoke John Baptist, Kilama Kamugisha, Gloria Akurut, Nigel Field, Lydia Franklinos, Naomi Fuller, Rory Gibbs, Sam Tweed, Janet Seeley, Laura Ferguson, Michelle Anderson, Sarah Emoto

**Affiliations:** 1 Department of Arbovirology, Emerging and Re-Emerging Infectious Diseases, Uganda Virus Research Institute (UVRI), Plot 51-59, Nakiwogo Road, Entebbe, Uganda; 2 National Animal Disease Diagnostics and Epidemiology Centre (NADDEC), Department of Animal Health, Ministry of Agriculture, Animal Industry and Fisheries (MAAIF), P. O. Box 513, Entebbe, Uganda; 3 MRC/UVRI & LSHTM Uganda Research Unit, Uganda Virus Research Institute (UVRI), Plot 51-59, Nakiwogo Road, Entebbe, Uganda; 4 Uganda Wildlife Authority, Kampala, Uganda; 5 College of Veterinary Medicine, Animal Resources and Biosecurity (CoVAB), Makerere University, Kampala, Uganda; 6 One Health Coordination Office (OHCO), National One Health Platform (NOHP), Ministry of Health, Kampala, Uganda; 7 Conservation Through Public Health (CTPH), Entebbe, Uganda

**Keywords:** Rift Valley fever virus, Seroprevalence, Small ruminants, Human-livestock-wildlife interface, Conservation areas

## Abstract

**Background:**

Following the first laboratory confirmed human Rift Valley Fever Virus (RVFV) outbreak in 48 years, Uganda has continued to detect sporadic outbreaks, particularly within the cattle corridor since 2016. Although wildlife potentially harbors RVFV strains, livestock exposure to RVFV at human-livestock-wildlife interfaces remains underexplored in major Ugandan conservation areas.

**Methods:**

A cross-sectional quantitative study was conducted between August, 2022 and March, 2023, at Satellite Research Sites (SRS), located in Bwindi-Mgahinga, Lake Mburo, Queen Elizabeth, Murchison Falls, and Pian Upe conservation areas in Uganda, which were selected for their high human-livestock-wildlife interactions. Using a two-stage sampling design, small ruminants were sampled from randomly selected herds within villages adjacent to the national parks. Blood samples were collected, and analysed with a validated in-house IgG indirect ELISA at the One Health Laboratory. ArcGIS Survey123 was used to capture the field data. Modified Poisson regression for binary outcomes and relative risks estimates were used.

**Results:**

A total of 1,690 small ruminants were sampled: 83.4% goats (caprine species), 16.6% sheep (ovine species). Of these, 92.4% were local breeds and 7.6% were exotic breeds. The females were 88.6% and 11.4% were males, with a mean age of 3 years. Overall RVFV seropositivity for both sheep and goats was 41.1% (695/1690), 95% CI (38.7–43.4%). Seropositivity per species was 42.4% (598/1409) in goats and 34.5% (97/281) in sheep. Exotic breeds and females had higher RVFV seroprevalence rates at 55.5% (71/128) and 41.8% (625/1,495) respectively. Modified Poisson regression analysis revealed that older animals (RR = 1.10, 95% CI: 1.06–1.14, *p* < 0.001) had higher risk of RVFV seropositivity, whereas local breeds (RR = 0.77, 95% CI: 0.63–0.93, *p* = 0.009) had reduced exposure risk to RVFV. Low RVFV exposure was evidenced in different management systems of grazing and watering the small ruminants.

**Conclusion:**

The high RVFV past exposure among small ruminants justifies the need for further studies to assess recent infections at the human-livestock-wildlife interface. Targeted interventions such as regulated park grazing, integrated vector control, and continuous surveillance should be implemented to minimize RVFV transmission in these high risk areas.

## Introduction

Rift Valley fever (RVF) is an emerging, mosquito-borne zoonotic disease that affects various ruminant species and humans. This disease is caused by Rift Valley fever virus (RVFV), a single-stranded RNA arbovirus belonging to the Phlebovirus genus in the Phenuiviridae family of the order Bunyavirales [1]. Rift Valley fever (RVF) remains a significant zoonotic disease in Africa and the Arabian Peninsula, with increasing seropositivity observed among both humans and livestock.

RVFV circulation has been documented across various regions in Africa. Serological surveys have documented RVFV in livestock in West Africa [2, 3], in South Africa [4], and Central and East Africa [5, 6]. The virus causes the death of young animals and abortion storms among gravid females, especially cattle, sheep, goats, and camels [7]. The epizootic that occurred in 11 regions in Tanzania and Kenya in 2007 resulted in the death of 16,973 cattle, 20,193 goats, and 12,124 sheep. Over 46,000 livestock also suffered abortions due to Rift Valley fever virus. This outbreak caused a loss of more than 2.1 billion Kenyan shillings (KSh) (equivalent to United States Dollars (USD) 32 million), and the total economic losses affecting livestock in East Africa exceeded USD 60 million [8].

The RVFV epidemiological and transmission cycle comprises mosquitoes, domestic, wild animals, humans and favourable climatic conditions, which encourage the breeding of the mosquitoes [9]. *Aedes* mosquitoes [10, 11] are the main vectors of the virus. *Culex* [11, 12] and *Anopheles* mosquitoes [12] have also been reported as secondary vectors of Rift Valley fever virus. Ticks [13, 14] have been documented as potential mechanical transmitters of RVFV from laboratory investigations. Ticks can also be infected with RVFV during feeding, during periods of high RVFV viremia in livestock and other animals [15].

Surrounding vegetation, poor soil drainage, and flat areas provide conducive environments for the breeding of mosquitoes [16].

Wildlife play a role in RVFV ecology. Antibodies have been detected in many wild animals in Africa, such as zebra, topi, impala [17], springbok, wildebeest, and black-faced impala [18]. While some wild animals exhibit mild disease, others suffer abortion or death [19], serving as sources of viral amplification and spillover to domestic animals and humans during epizootic periods [20].

Since the virus was first isolated from a sheep flock in Kenya in 1930 [21], many studies have reported its seroprevalence among livestock and humans across Africa. Livestock outbreaks in East Africa have been linked to periods of heavy rainfall and flooding, which facilitate vector multiplication [22].

**Fig. 1:**
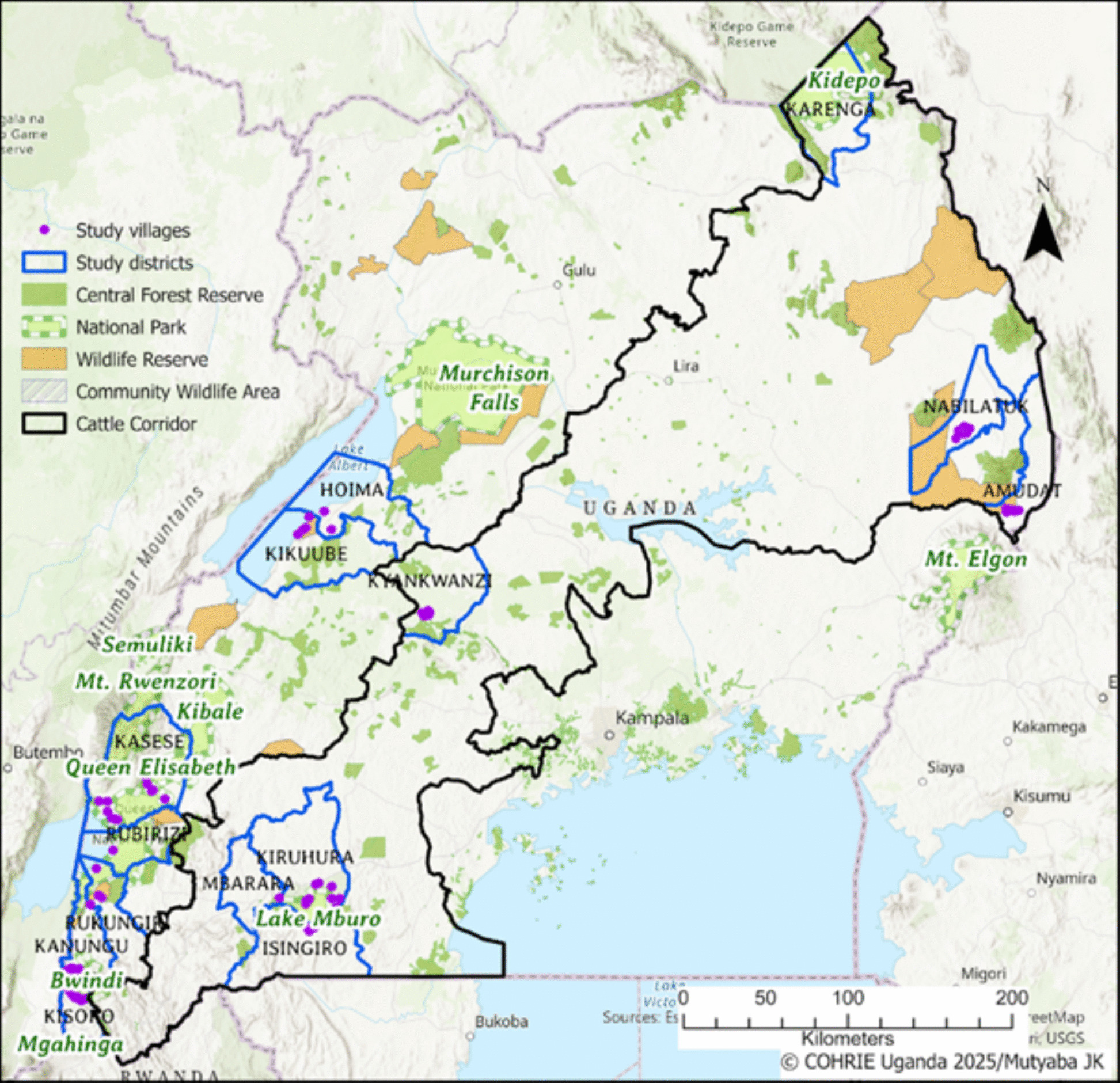
Map showing the study areas where the small ruminants were sampled for RVFV seroprevalence, between August, 2022-March, 2023. © COHRIE-Uganda

In Uganda, RVF is recognized as one of the seven major zoonotic diseases of public health importance because of its epidemic potential and serious impact on humans [23]. Several outbreaks have been reported, especially in districts within the cattle corridor [24, 25]. The cattle corridor is a semi-arid transition zone across the centre of the country, between the wet forest/grassland mosaics to the south on the Uganda-Tanzania border around Lake Victoria, and the arid grasslands on the Uganda-Kenya-Sudanese border in the north east in the Karamoja area [26], as shown in (Fig. 1).

More recent studies have reported RVFV circulation within districts bordering Uganda’s conservation areas. According to Uganda Wildlife Act (Chapter 315), a Conservation Area (CA) is wildlife protected area (i.e. national parks, game reserves) and a community wildlife management area or any other area declared for purposes of protection of vulnerable fauna and flora and vested for management partly by local communities [27].

Findings from a national serosurvey indicated an overall RVFV seroprevalence of 11.3%, with 15% in cattle, 5.6% in goats and 10.9% in sheep [28]. Despite these findings, specific data on RVFV seroprevalence among livestock at the human-livestock-wildlife interface remain scarce.

Although RVFV antibodies have been isolated from wildlife samples [17, 18, 29], in Uganda, Birungi et al*.* [17] explored the ecological interactions among wildlife, livestock, and humans in conservation areas. The interface between these groups in conservation areas likely presents unique transmission dynamics due to the close interactions between wildlife, livestock, and human populations; however, the risk factors specific to these regions remain poorly understood.

No effective control strategies against RVF in Uganda have been suggested, despite the potential of this disease to cause large scale outbreaks in humans or epizootics in susceptible animal populations. The infection threatens not only livestock production and livelihoods but also poses significant public health risks [23]. A study assessing the 2006–2007 outbreak estimated the public health impact of RVFV in terms of disability-adjusted life years (DALYs) at 3.4 DALYs per 1000 people in the family and cost approximated to KSh 10,000 per person for case management, equivalent to USD 118 [20].

Therefore, this study aimed to determine the seroprevalence of RVFV and describe the associated risk factors among small ruminants at selected sites with high human-livestock-wildlife interactions within Uganda’s wildlife conservation areas. These small ruminants (sheep and goats), play a critical role in rural livelihoods. These animals play a significant economic and nutritional role in rural households, but are often underrepresented in surveillance programs [16]. They are often maintained in extensive systems with minimal vector protection, increasing their exposure to arboviruses. Previous studies have shown high RVFV seroprevalence rates among small ruminants: 25.8% in Tanzania [5], 35.8% in Mozambique [30], and 29.7% in sheep in South Africa [31], emphasizing their vulnerability. Moreover, small ruminants often serve as early indicators of RVFV outbreaks because of their sensitivity to infection and associated clinical signs such as abortion and sudden death. By focusing on small ruminants, we sought to fill critical knowledge gaps and inform targeted control strategies for RVFV in Uganda's high-risk areas.

## Methods

### Study area

This study was carried out in 13 districts within selected conservation areas in the cattle corridor of Uganda (Fig. 1), between August, 2022 and March, 2023. The cattle corridor, which hosts a large population of Uganda’s livestock, covers approximately 35% of the country and extends from the southwest to the northeastern region of the country [26]. The study was focused in areas with the greatest human-livestock-wildlife interaction known as Satellite Research Sites (SRS) in our study, in the following districts; Kanungu and Kisoro in the Bwindi-Mgahinga Conservation Area (BMCA), Kiruhura, Isingiro, and Mbarara city in the Lake Mburo-Nakivale Conservation Area (LMCA), Hoima, Kikuube, and Kyankwanzi districts in the Murchison Falls Conservation Area (MFCA), Nabilatuk and Amudat districts in the Pian-Upe Conservation Area (PUCA), and Rukungiri, Kasese and Rubirizi districts in the Queen Elizabeth Conservation Area (QECA).

### Study design, sample size determination, and sample and data collection

We conducted a cross-sectional quantitative study within SRS in the study conservation areas. Households in these SRSs were mapped prior to sampling to minimize bias during sample collection, as described by Kizito et al*.* [32].

The sample size was calculated using the formula by Thrusfield et al*.* [33]. We computed the minimum number of samples to collect from every conservation area using a previous RVFV seroprevalence of 11.3% reported in a study by Tumusiime et al*.* [28]; the margin of error was 0.05, and the confidence interval was 95%. A design effect of 2 was also used to calculate the sample size given the nature of livestock sampling within herds. The minimum number of samples to be collected from each conservation area was 308. Our calculated minimum sample size (n _min_) was 1,540 livestock. A two-stage sampling design was used to sample the small ruminants; stage one involved the selection of villages within the SRS. The villages were selected on the basis of their proximity to the national parks, where by the closest were selected within the SRS. Stage two involved random selection of herds from these villages selected in Stage one. The herd inclusion criteria that were followed in this study were; location of the herd in an area that was within a 5 km radius of the national park within the cattle corridor, as this was assumed as the maximum distance the herds would be moved from the home steads to the national parks. The herds also had to be in an area with a history of zoonotic disease outbreaks in these conservation areas. Only herds whose owners consented to the study were sampled [32]. A maximum of 30 animals were randomly sampled from a herd that was selected for the study to minimize selection bias. The response rate for this study was 109.7% (1,690/1,540) which resulted in a total number of livestock that were sampled from all the study conservation areas of 1,690. This occurred because of the proper mobilization and willingness of the farmers to participate in the study.

Restraint was performed by the herdsmen on these farms. Blood (5–10 ml) was drawn from individual animals' jugular or coccygeal veins into SST vacutainer tubes, using vacutainer needles, and needle holders. Blood samples were collected by trained veterinarians. Each sample was uniquely identified by pre-labeled stickers defining the CA and individual animal number for ease of analysis. After collection, samples were placed on ice and transported to the Regional Veterinary Laboratories for aliquoting and temporary storage at 4–8 °C within 6–12 h; later they were transported to the One Health Laboratory at the Uganda Virus Research Institute (UVRI) and stored at −20 °C before further analysis.

A data collection questionnaire based on previous RVFV studies, and selected study areas was digitized and deployed on tablets (Samsung A 8 SM-X205) that had the ArcGIS Survey123 software application (Environmental Systems Research Institute (Esri Eastern Africa)) installed, and was used to systematically capture relevant information during the sampling process. Prior to data and sample collection, location information was instantly recorded as GPS coordinates with an accuracy of 8–10 m to ensure and control spatial data quality. Information pertaining to herd characteristics, such as the number of animals, grazing patterns, watering methods, vaccination history, and treatment records, was also gathered. Furthermore, individual animal data, including age which was determined by using the tooth eruption method; sex, breed, and origin; and various physiological parameters, such as temperature, demeanor, color of mucous membranes, fecal color and consistency, as well as any pertinent medical history, were recorded and saved via the data collection tool. Additionally, any notable observations such as wounds or the animal's location during sample collection were documented for analysis.

### Blood sample analysis

Before analysis, the serum samples were sorted by species and organized into species-specific worksheets to streamline processing and prevent omission. RVFV in-house indirect ELISA was used to detect the presence of IgG anti-RVFV antibodies. IMMULON® 4HBX (Thermofisher scientific_ Flat Bottom Micro titer®) ELISA plates were coated with recombinant RVFV nucleoprotein antigen (Ref: DAG-WT641, Creative Diagnostics®), which was diluted in 1X phosphate buffered saline (PBS) solution, to a concentration of 1 µg/ml per plate for all the species. Fifty microliters of the diluted antigen were added to each well of the ELISA plates and stored in the refrigerator at 4–8^0^C overnight. The samples were diluted with 5% skimmed milk at a dilution ratio of 1:200 for the goat samples, and 1:25 for the sheep samples. Known positive and negative controls per species obtained from the National Animal Disease Diagnostics and Epidemiology Centre-Ministry of Agriculture Animal Industry and Fisheries (NADDEC-MAAIF) laboratory, were also diluted at the ratios used for the samples. The ELISA plates were first washed three times to remove excess uncoated antigen using wash buffer (1X PBS and 0.1% Tween 20), and then bloated. The plates were blocked with 200 µl (per well on the ELISA plate) of 5% skimmed milk in wash buffer and incubated for 1 h at 37^o^ C. Fifty microliters of the diluted samples and controls were loaded in duplicate on washed ELISA plates and incubated for 1 h. The species-specific conjugates were diluted with 5% skimmed milk in wash buffer at a ratio of 1: 24,000 for sheep samples (rabbit anti-sheep IgG polyclonal antibodies; Ref: DPAB21850-H, Creative Diagnostics®, NY-USA) and 1: 15,000 for goat samples (rabbit anti-goat IgG polyclonal antibodies; Ref: DPAB21576-H, Creative Diagnostics®, NY-USA); 100 µl of the conjugate was added per well and incubated for 1 h at 37^o^ C. The plates were washed 5 times with wash buffer to remove any excess unbound conjugate. Fifty microliters of 3,3^’^,5,5^’^- tetramethylbenzidine (TMB) ELISA peroxidase substrate was added to each well and then incubated at room temperature for 15 min in a dark room. Hydrochloric acid was added after 15 min to stop the reaction. The ELISA plates were then read using GEN5 software (Gen5™ Microplate Reader and Imager Software) on Biotek ELx 808, USA to obtain the optical densities. The results were exported to Microsoft Excel 2010, and interpreted using the following formula [Mean negative control + (3*Standard Deviation of Negative control)] by Lardeux et al*.* [34] to get the cut-off value.

### Data analysis

Both field and laboratory data were imported into STATA version 18 (StataCorp, College Station, TX) software for further analysis. The analysis was guided by the study objectives, which were to determine the seroprevalence of RVFV among small ruminants and to describe the risk factors associated with exposure in areas with highest human-livestock-wildlife interactions within conservation areas. The data were analysed using both descriptive and inferential statistical methods.

All the animal-level data including age, sex, and species were captured in the database using factor variables. Herd characteristics were also entered and analysed as factor variables. Both animal and herd variables were recorded as independent variables, while RVFV exposure was the dependent variable during the analysis.

The number of small ruminants screened by conservation area, species, age, sex, breed, and origin were determined using descriptive statistics. The data were presented as frequencies and percentages. The overall RVFV seroprevalence among all the livestock sampled and its confidence interval (CI) were determined.

The outcome variable, RVFV seropositivity, was treated as binary (positive/negative). To identify factors associated with seropositivity, modified Poisson regression with robust standard errors was used to estimate risk ratios (RRs) and their corresponding 95% CI. Initially, crude (univariate) modified Poisson regression models were fitted to assess the relationship between each independent variable and RVFV seropositivity. Variables with a *p*-value < 0.20 in the crude analysis were considered for inclusion in the multivariate modified Poisson regression model (Table 3), which was used to adjust for potential confounders.

The final adjusted model included key covariates such as age, conservation area, and herd size, reflecting the study aim of identifying risk factors for RVFV exposure. All models produced RRs with 95% CIs, and statistical significance was determined at a *p*-value threshold of < 0.05. Model diagnostics and multicollinearity checks were performed prior to final model selection.

## Results

### Livestock demographics and characteristics

A total of 1,690 small ruminants were sampled from 5 conservation areas including; 1,555 (92.4%) local breeds of small ruminants, 1409 (83.3%) goats, 281 (16.6%) sheep, and 1495 (88.6%) females, and the mean age for the sampled small ruminants was 3 years, as shown in Table 1.

**Table 1:** Demographics of the small ruminants sampled from all the conservation areas between August, 2022-March, 2023

Variable	Category	Frequency; n (%) (*n* = 1690)
Age	Mean (SD)	3.24 (1.53)
Sex*	Female	1,495 (88.6%)
	Male	192 (11.4%)
Origin*	Born in herd	1,598 (96.9%)
	Brought in	51 (3.1%)
Conservation Area*	Bwindi Mgahinga CA	386 (22.9%)
	Lake Mburo CA	362 (21.4%)
	Murchison Falls CA	103 (6.1%)
	Pian Upe CA	340 (20.1%)
	Queen Elizabeth CA	497 (29.4%)
Breed*	Exotic	128 (7.6%)
	Local	1,555 (92.4%)
Species	Goats	1,409 (83.4%)
	Sheep	281 (16.6%)

^*^Missing data: sex (3), origin (50), conservation area (2), breed (7)

### RVFV seropositivity

The overall RVFV seropositivity among the small ruminants from all the conservation areas was 41.1% (695/1690: 95% CI 38.7–43.4%), (Table 2)**.**

**Table 2:** RVFV seroprevalence among the sheep and goats sampled from all conservation areas between August, 2022-March, 2023

FACTOR	LEVEL	RVFV		*p*-value
		**NEGATIVE (*N* = 995)**	**POSITIVE (*N* = 695)**	
Age	Mean (SD)	3.25 (1.53)	3.23 (1.53)	0.860
Sex	Female	870 (58.2%)	625 (41.8%)	0.120
	Male	123 (64.0%)	69 (36.0%)	
Breed	Exotic	57 (44.5%)	71 (55.5%)	0.001
	Local	933 (60.0%)	622 (40.0%)	
Species	Goats	811 (57.6%)	598 (42.4%)	0.014
	Sheep	184 (65.5%)	97 (34.5%)	

^*^Missing data: sex (3), breed (7)

### Risk factors associated with RVFV occurrence among small ruminants sampled from the study conservation areas between August, 2022-March, 2023

Modified Poisson with robust standard errors was applied to the dataset where Relative Risks (RR) were computed for RVFV exposure. Table 3 shows that animals sampled from LMCA, MFCA, PICA, and QECA had higher risk of RVFV exposure compared to those sampled from BMCA (*p* < 0.001). Older animals had a higher risk of past exposure to RVFV compared to young ones (*p* < 0.001). Compared with exotic breeds, local breeds had a lower risk of exposure to RVFV (*p* < 0.001). Herd variables show that animals grazed in communal grazing grounds, farms, and zero grazing units had lowered risk of exposure compared to those grazed in both parks and farms (*p* < 0.001). Animals watered in rivers, streams, lakes, piped water, valley dams, valley dams and tanks had lowered risk of RVFV exposure compared to those watered with hand fetched water (*p* < 0.001). Herds grazed in the parks in the dry season had less risk of RVFV exposure compared to those grazed throughout the year (*p* = 0.002).

**Table 3:** Showing the Crude and adjusted analysis of the small ruminants sampled from all the conservation areas in Uganda between August, 2022-March, 2023

FACTOR	LEVEL	CRUDE ESTIMATES		ADJUSTED MODEL	
		**RR (95% CI)**	***p*-value**	**RR (95% CI)**	***p*-value**
Age*	Per year increase	0.99 (0.96; 1.03)	0.860	1.10 (1.06; 1.14)	< 0.001
Sex	Female	Reference	0.135		0.077
	Male	0.85 (0.70; 1.04)		0.83 (0.68; 1.01)	
Breed	Exotic	Reference	< 0.001		0.009
	Local	0.72 (0.61; 0.85)		0.77 (0.63; 0.93)	
Origin	Born in herd	Reference	0.400		0.217
	Brought in	0.85 (0.58; 1.23)		0.78 (0.53; 1.15)	
Conservation Area *	Bwindi Mgahinga	Reference	< 0.001		< 0.001
	Lake Mburo	2.22 (1.74; 2.83)		2.28 (1.79; 2.91)	
	Murchison Falls	1.74 (1.22; 2.47)		1.89 (1.32; 2.69)	
	Pian Upe	1.72 (1.32; 2.24)		1.78 (1.34; 2.36)	
	Queen Elizabeth	3.64 (2.92; 4.53)		4.16 (3.34; 5.18)	
Herd size*	Per unit increase	1.00 (0.99; 1.001)	0.510	0.99 (0.99; 1.00)	< 0.001
Park grazing	No	Reference	< 0.001		0.324
	Yes	1.54 (1.29; 1.84)		1.16 (0.86; 1.57)	
Park frequency	Always	Reference	< 0.001		0.002
	Occasionally	1.14 (0.88; 1.47)		1.32 (0.96; 1.80)	
	Seasonally (dry)	0.66 (0.51; 0.84)		0.69 (0.52; 0.91)	
Grazing pattern	Both farm & park	Reference	< 0.001		0.001
	Communal	0.74 (0.54; 0.99)		0.75 (0.54; 1.06)	
	Farm	0.44 (0.31; 0.62)		0.57 (0.39; 0.81)	
	Park	0.72 (0.51; 1.02)		0.95 (0.67; 1.36)	
	Zero	0.62 (0.39; 0.99)		0.83 (0.42; 1.64)	
Watering methods	Hand fetched	Reference	< 0.001		< 0.001
	Lake	1.01 (0.84; 1.22)		0.75 (0.63; 0.91)	
	Piped water	1.00 (0.81; 1.25)		0.89 (0.74; 1.08)	
	River	0.66 (0.53; 0.81)		0.88 (0.71; 1.09)	
	Stream	1.04 (0.84; 1.29)		0.88 (0.72; 1.07)	
	Valley dam	0.16 (0.07; 0.38)		0.29 (0.14; 0.63)	
	Valley dam/tank	0.56 (0.45; 0.69)		0.53 (0.42; 0.68)	
Clinical signs (present)	Ocular discharge	0.81 (0.26; 2.51)	0.716	1.10 (0.42; 2.85)	0.842
	Nasal discharge	1.24 (1.02; 1.51)	0.028	1.01 (0.85; 1.20)	0.898
	Oral lesions	(2.62e-16) **	< 0.001	(7.53e-7) **	< 0.001
	Abnormal breathing	1.25 (1.02; 1.54)	0.027	1.02 (0.85; 1.23)	0.755
	Abnormal faeces	0.78 (0.46; 1.30)	0.346	0.80 (0.48; 1.34)	0.406
	Abnormal walking	0.67 (0.31; 1.42)	0.299	0.57 (0.25; 1.31)	0.189
	Other	0.69 (0.54; 0.88)	0.003	0.74 (0.57; 0.97)	0.035

^*^The adjusted analysis for the small ruminants’ model included the variables; age, conservation area, and herd size. Specifically, age and herd size were included a priori because they are known potential confounders in livestock disease risk. Conservation area was added based on statistical criteria showing a strong crude association with the outcome and met the inclusion threshold for adjustment

^**^ RR-very small; very few animals with visible oral lesions

Further analyses were done for the sex, origin of the animal, any clinical signs present and herd size.

## Discussion

The overall RVFV seroprevalence among livestock from all the conservation areas was reported to be 41.1% (695/1690) (Table 2). This finding provides evidence of RVFV past exposure among small ruminants in communities surrounding within the conservation areas in Uganda. Surveys conducted in other African regions have shown varying RVFV seroprevalence rates. For instance, Bergh et al*.* [4] reported a seroprevalence of 31.7% in goats in KwaZulu-Natal, South Africa, while Sindato et al*.* [5] reported seroprevalences of 29.7% in sheep, and 22.0% in goats in Tanzania. A recent survey in northern Senegal by Mhamadi et al*.* [3] reported RVFV seroconversion of 66.7% among sheep. These varying seroprevalences reflect considerable regional heterogeneity in RVFV exposure among small ruminants. The high exposure observed in this study likely reflects the selection of sites characterized by intense human-livestock-wildlife interactions and ecological conditions that favor mosquito increase in and around these conservation areas. Secondly, the higher seroprevalence reported in this study could be attributed to differences in the serological assays employed. For instance, a similar study conducted in Uganda using a validated in-house protocol reported RVFV exposure rates of 2.6% among goats and 2.0% among sheep sampled nationwide [35]. In contrast, another study using a commercial competition multispecies anti-RVFV IgG ELISA assay found seroprevalences of 5.6% among goats and 10.9% among sheep sampled from various districts in Uganda [28].

Areas within BMCA are characterized by higher slopes and altitudes, which likely contributed to a lower risk of RVFV exposure among the small ruminants sampled compared to those from lower-lying regions in other wildlife conservation areas. Low-lying, flat terrains are more prone to seasonal flooding after heavy rainfall, creating ideal breeding habitats for mosquitoes [36, 37]. Similarly, factors such as a high topographic wetness index, gentle land slopes, and water-retentive soils have been statistically associated with increased RVFV seroprevalence in livestock [28, 38]. In Kenya’s Baringo region, areas with slopes of less than 6% and poorly drained soils were found to support numerous mosquito larval populations than steeper, well-drained zones [39].

According to our study, herds that grazed in both parks and farms had a significantly higher risk of exposure to RVFV compared to those under other grazing patterns such as communal grazing, farm-only, or zero grazing (Table 3). During field sampling, domestic animals were frequently observed sharing grazing grounds with wildlife, creating opportunities for virus spillover. Similar findings were reported by Birungi et al*.* [17], who observed close interactions between domestic animals and wildlife in the grazing areas of Lake Mburo National Park, suggesting possible RVFV transmission between the two populations. This practice of grazing within the park commonly occurs during the dry season when pastures and water are scarce on farmlands. However, contrary to the initial crude analysis, grazing within national parks was not independently associated with RVFV exposure in the adjusted model (RR = 1.16, *p* = 0.324). Interestingly, seasonal (dry-season) park grazing appeared to be protective (RR = 0.69, *p* = 0.002), possibly due to reduced vector activity during dry conditions. These findings suggest that while RVFV exposure can occur within park environments, the actual risk is influenced by multiple interacting factors such as seasonality, park location, and herd management practices.

Additionally, our findings revealed that goats had a higher risk of RVFV seropositivity than sheep (Table 4). This difference is likely linked to their feeding behavior, as goats tend to feed on shrubs and vegetation commonly found in park and farm environments that are habitats for mosquitoes and other mechanical vectors of RVFV.

Our study found that older animals had a higher risk of past exposure to RVFV compared to younger animals (Table 3). This is likely due to cumulative lifetime exposure to infected mosquito bites, as well as the long-term persistence of RVFV-specific IgG antibodies following previous infections. Additionally, exotic breeds showed greater exposure than local breeds, which may be explained by their lower immunological adaptation to endemic pathogens and potential differences in innate or adaptive immune responses under tropical, vector-rich conditions. These findings align with previous studies, including Isidore et al*.* [40], who reported higher RVFV seroprevalence among exotic cattle in Rwanda, and Nyakarahuka et al*.* [35], who observed similar trends in Uganda, highlighting age and breed as important factors associated with increased RVFV exposure.

Water sources were found to significantly influence RVFV risk in our study (Table 3). Animals watered from hand-fetched shallow sources exhibited higher RVFV seropositivity compared to those accessing lakes, piped water, rivers, streams, valley dams, or tanks. Our field observations indicated poor hygiene around goat pens, with water placed in shallow troughs and basins that likely remained for extended periods. These shallow containers and puddles can serve as mosquito breeding sites, particularly where water continuously spills onto the ground, creating stagnant pools [36, 37]. Supporting this, a geospatial analysis in Uganda found higher RVFV seroprevalence in areas characterized by abundant standing water and stable precipitation [28]. Similarly, in nomadic pastoralist settings, herdsmen identified watering points as key zones for mosquito bites, emphasizing the importance of water access behaviors in RVFV transmission dynamics [16, 41].

### Limitations of the study

Our study was unable to report on current RVFV exposure in the study areas, as the primary focus was on seroprevalence resulting from past infections. The in-house assay used was specific for IgG antibody detection, which does not differentiate between recent and historical exposure. Attempts to detect viral RNA using RT-PCR yielded negative results, likely because the sampled animals were asymptomatic and there was no active RVF outbreak in the study areas.

Additionally, farmers within the selected sampling areas did not maintain written records regarding the origin of the animals sampled. Information reported in this study was therefore based on verbal accounts provided by farmers and herdsmen, which may have introduced recall bias or limited the accuracy of some background data.

## Conclusion

Our study highlights RVFV seropositivity among small ruminants sampled at high human-livestock-wildlife interface. We recommend further investigations to quantify recent RVFV exposure variations in small ruminants and to assess the influence of environmental factors such as altitude and slope within conservation areas. Farmers should be sensitized to avoid grazing livestock within national parks to minimize the risk of spillover infections from wildlife. Integrated vector control programs targeting both mosquitoes and ticks should be prioritized, with emphasis on the use of appropriate acaricides, such as permethrins, for effective tick and insect control. Furthermore, strengthening RVFV surveillance systems, introducing vaccination programs, and conducting anthropological studies to better understand human-livestock-wildlife interactions are critical steps toward preventing future outbreaks in areas of high human-livestock-wildlife interfaces in Uganda’s wildlife conservation areas.

Despite the study’s limitations, the findings remain epidemiologically significant as they reveal areas of sustained viral exposure and potential endemicity. These insights provide an important foundation for the development of targeted control and prevention strategies against RVFV infections in Uganda’s human-livestock-wildlife interfaces.

## Data Availability

All the data and material of this study are available upon request to the corresponding author including all field and laboratory original data on reasonable request.

## Abbreviations

BMCA: Bwindi-Mgahinga Conservation Area
CCHFV: Crimean Congo Haemorrhagic fever virus
COHRIE: Collaborative One Health Research Initiative on Epidemics
DHIS 2: District Health Information System 2
ELISA: Enzyme Linked Immuno Sorbent Assay
GIS: Geographical Information System
IgG: Immunoglobulin G
IgM: Immunoglobulin M
RR: Relative Risk
KSh: Kenyan shillings
LMCA: Lake Mburo-Nakivale Conservation Area
MAAIF: Ministry of Agriculture, Animal Industry and Fisheries
MFCA: Murchison Falls Conservation Area
OH laboratory: One Health Laboratory
PUCA: Pian-Upe Conservation Area
QECA: Queen Elizabeth Conservation Area
QGIS: Quantum Geographical Information System
RNA: Ribonucleic acid
RT-PCR: Reverse Transcriptase Polymerase Chain Reaction
RVFV: Rift Valley fever virus
SRS: Satellite Research Sites
SST: Serum Separating Tube
UNCST: Uganda National Council for Science and Technology
USD: United States Dollar
UVRI: REC- Uganda Virus Research Institute- Research Ethics Committee
UVRI: Uganda Virus Research Institute
